# Navigating SARS-CoV-2-related immunopathology in Crohn’s disease: from molecular mechanisms to therapeutic challenges

**DOI:** 10.1186/s12985-024-02529-1

**Published:** 2024-11-13

**Authors:** Chang-Cyuan Chen, Yu-An Lin, Kuan-Ting Liu, Chun-Yao Huang, Chun-Ming Shih, Yuan-Ti Lee, Jun-Liang Pan, Ai-Wei Lee

**Affiliations:** 1 Department of Anatomy and Cell Biology, School of Medicine, College of Medicine, Taipei Medical University, Taipei, 11031 Taiwan; 2 Department of Medical Education, Chung Shan Medical University Hospital, Taichung, Taiwan; 3 Department of General Medicine, Chang Gung Memorial Hospital, Taipei Medical University, Taipei, 11031 Taiwan; 4 Department of Internal Medicine, School of Medicine, College of Medicine, Taipei Medical University, Taipei, 11031 Taiwan; 5 Cardiovascular Research Center, Taipei Medical University Hospital, Taipei, 11031 Taiwan; 6 Taipei Heart Institute, Taipei Medical University, Taipei, 11031 Taiwan; 7 School of Medicine, Chung Shan Medical University, Taichung City, 40201 Taiwan; 8 Division of Infectious Diseases, Department of Internal Medicine, Chung Shan Medical University Hospital, Taichung City, 40201 Taiwan; 9 Division of Gastroenterology and Hepatology, Department of Internal Medicine, Taipei Medical University Hospital, Taipei, 11031 Taiwan

**Keywords:** Crohn’s disease, SARS-CoV-2, Immunosuppressive treatment

## Abstract

Severe acute respiratory syndrome coronavirus 2 (SARS-CoV-2) not only posed major health and economic burdens to international societies but also threatens patients with comorbidities and underlying autoimmune disorders, including Crohn’s disease (CD) patients. As the vaccinated population is gradually relieved from the stress of the latest omicron variant of SARS-CoV-2 due to competent immune responses, the anxiety of CD patients, especially those on immunosuppressive treatment, has not subsided. Whether the use of immunosuppressants for remission of CD outweighs the potential risk of severe coronavirus disease 2019 (COVID-19) has long been discussed. Thus, for the best benefit of CD patients, our primary goal in this study was to navigate the clinical management of CD during the COVID pandemic. Herein, we summarized COVID-19 outcomes of CD patients treated with immunosuppressive agents from multiple cohort studies and also investigated possible mechanisms of how SARS-CoV-2 impacts the host immunity with special consideration of CD patients. We first looked into the SARS-CoV-2-related immunopathology, including lymphocytopenia, T-cell exhaustion, cytokine storms, and their possible molecular interactions, and then focused on mechanistic actions of gastrointestinal systems, including interruption of tryptophan absorption, development of dysbiosis, and consequent local and systemic inflammation. Given challenges in managing CD, we summarized up-to-date clinical and molecular evidence to help physicians adjust therapeutic strategies to achieve the best clinical outcomes for CD patients.

## Introduction

Severe acute respiratory syndrome coronavirus 2 (SARS-CoV-2), a newly identified beta-coronavirus first discovered in Wuhan, China, accounted for severe respiratory symptoms and was subsequently named coronavirus disease 2019 (COVID-19) by the World Health Organization (WHO) [1]. The outbreak of SARS-CoV-2 dramatically influenced a variety of industries across nations, causing an estimated healthcare loss of US$200 billion in the United States and a global economic loss of US$2 trillion [2]. On top of the economic damage, this SARS-CoV-2 pandemic also influenced clinical management, especially for those of an older age, and those with comorbidities and chronic health concerns [3].

Crohn’s disease (CD), one of the inflammatory bowel diseases (IBDs), is characterized by ulceration, erythema, and mucosal edema in discontinuous areas within the intestines [4]. Steroids, immunomodulators, and biologics are widely used to suppress intestinal immune responses, but close monitoring is necessary for better prognoses and clinical outcomes [4]. Discontinuation of immunosuppressive treatment may lead to increased risks of relapse and complications [5]. To achieve better clinical remission, close monitoring and timely adjustment of therapeutic plans are crucial for managing CD.

Unfortunately, the outbreak of SARS-CoV-2 further complicated the clinical management of CD. Several studies showed that administering immune suppressants can increase the risk of opportunistic viral infections [6] and may be associated with severe COVID-19 outcomes [7]. Therefore, the American Gastroenterological Association (AGA) suggested that CD patients postpone their immunosuppressive therapy until resolution of COVID-19 to avoid unexpected risks of infection and severe clinical outcomes [8]. However, several other studies suggested that immunosuppressants may be beneficial to patients infected with COVID-19 by mitigating cytokine storms, such as anti-interleukin (IL)-6 antibodies [9] and anti-tumor necrosis factor (TNF) therapy [10]. Obviously, unraveling the mechanisms of how SARS-CoV-2 impacts host immunity is an unmet need.

In this review, we first examined how SARS-CoV-2 compromises immune systems by causing lymphocytopenia, T-cell exhaustion, and cytokine storms. Furthermore, we propose a possible mechanism of how SARS-CoV-2 aggravates gut inflammation in CD patients by interrupting the absorption of tryptophan and alters the composition of the gut microbiota. Last, multiple cohort studies were reviewed to assess better strategies for clinically managing CD patients during the COVID-19 pandemic.

## Impacts of SARS-CoV-2 infection on the immune system of patients

### Impacts on non-CD patients

#### Clinical features and cell entry of SARS-CoV-2

SARS-CoV-2, SARS-CoV, and the Middle East respiratory syndrome coronavirus (MERS) all belong to the *Betacoronavirus* genus and share genetic homology [11]. Clinical manifestations of patients infected with SARS-CoV-2 can range from mild respiratory symptoms encompassing fever, cough, and dyspnea to severe acute respiratory syndrome (SARS) [12]. Among all patients with SARS-CoV-2 infection, 80% are asymptomatic [13], whereas 80% of symptomatic patients present with mild symptoms, including fever, cough, and fatigue [14]. Nonetheless, severe symptoms not only occur in the lungs, such as hypoxia and pulmonary infiltration, but also involve the liver and digestive system, with elevated levels of liver enzymes [15] and gastrointestinal (GI) symptoms.

The entry of SARS-CoV-2 into the body is dependent on expression of angiotensin-converting enzyme 2 (ACE2) on cellular surfaces. After cleavage and activation by transmembrane serine protease 2 (TMPRSS2), spike proteins of SARS-CoV-2 recognize ACE2 as a receptor, which facilitates entry of the virus into cells [16]. It is not surprising that high ACE2 expression is found in type 2 pneumocytes [16], which may be attributed to the presence of a viral infection. Nonetheless, emerging studies revealed that ACE2 is also found in the oral and nasal mucosa, cholangiocytes, hepatocytes, cardiocytes, renal epithelial cells, and enterocytes, which may explain the emerge of extrapulmonary symptoms [17–21].

#### Lymphocytopenia in patients with SARS-CoV-2

Both innate and adaptive immune responses are activated when confronting a SARS-CoV-2 infection. First, delayed secretion of antiviral interferons (IFNs) triggered by rapid viral replication stimulates activation of macrophages [22]. Subsequently, IFN-induced activated macrophages recruit other inflammatory cells, including neutrophils, lymphocytes, natural killer (NK) cells, and dendritic cells [22]. Activated T cells and NK cells not only participate in cytotoxic actions of infected cells but also sustain activation of macrophages by secreting TNF, IFN-γ, and granulocyte-[monocyte/macrophage?] colony-stimulating factor (GM-CSF), which in turn activates more immune cells [22].

It is not surprising that lymphocyte counts increase after a viral infection, but a decreased lymphocyte count, or lymphocytopenia, was also found in severe SARS-CoV-2 patients. During the acute stage of SARS-CoV-2 infection, serum levels and activities of both cluster of differentiation 4-positive (CD4 +) T cells and CD8 + T cells had increased by 7 days after the onset of symptoms [23]. Nonetheless, decreased numbers of CD4 + T cells, CD8 + T cells, and NK cells were found in severely ill patients and in patients who died of COVID-19 [12, 24]. Furthermore, the extent of lymphocytopenia was suggested to be a predictor of clinical prognoses of SARS-CoV-2 infection. Percentages of lymphocytes to leukocytes were documented to have dropped below 5% in 11 patients who died of SARS-CoV-2 infection, whereas the lymphocyte percentage decreased less dramatically and had even increased by 10% and 20%, respectively, in patients with severe and moderate symptoms before they were discharged [25].

**Fig. 1:**
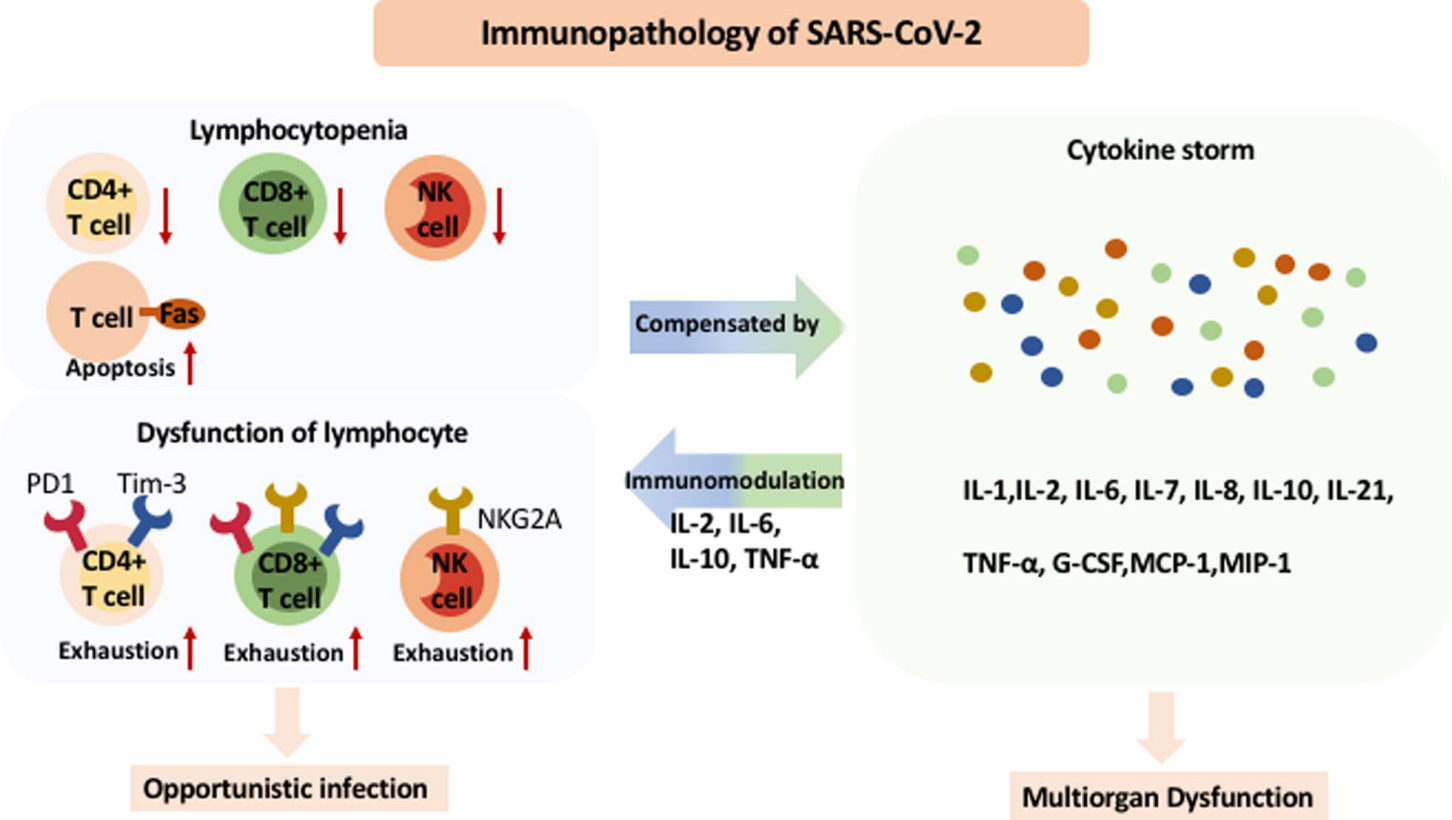
Immunopathology of severe acute respiratory syndrome coronavirus 2 (SARS-CoV-2)

The mechanism of lymphocytopenia in patients with SARS-CoV-2 is not well understood. We hereby discuss two possible reasons: T-cell apoptosis and immunomodulation by cytokines. On one hand, T-cell apoptosis can be attributed to lymphocytopenia, which was previously reported in MERS-CoV [26]. Higher levels of apoptotic markers, including CD95/Fas receptors on T cells and soluble FasL in plasma, and caspase-mediated T-cell apoptosis were correlated with lymphopenia and disease severity in COVID-19 patients [27]. On the other hand, the suppressive effect of cytokines should also be considered. TNF-α promotes T-cell apoptosis by binding to TNF receptor 1, while IL-2 hinders the function and proliferation of T cells by binding to the soluble IL-2 receptor (IL-2R), or CD25 [28]. Both serum levels of TNF-α and IL-2R are remarkably higher in COVID-19 patients with severe symptoms [29] (Fig. 1).

#### Cytokine storms in patients with SARS-CoV-2

Seemingly contradictory to lymphocytopenia, hypercytokinemia was observed in patients infected with SARS-CoV-2, as well as with SARS-CoV and MERS-CoV [30–32]. Cumulative studies discovered that a variety of cytokines, including IL-1, IL-6, IL-7, IL-8, IL-10, IL-21, TNF-α, granulocyte colony-stimulating factor (G-CSF), chemoattractant protein (MCP)-1, and macrophage inflammatory protein (MIP)-1α are involved in the inflammatory response against SARS-CoV-2 infection [12, 33] (Fig. 1).

To explain the coexistence of lymphopenia and hypercytokinemia in COVID-19, Diao et al. suggested that the source of IL-6, IL-10, and TNF-α was innate immune cells such as macrophages and monocytes rather than T cells and that these cytokines promote apoptosis of T cells and lead to lymphocytopenia in SARS-CoV-2 patients [34]. The decrease in lymphocytes abrogates the ability to tamper with innate immune responses and thus leads to dramatic innate immune responses and cytokine storms [35]. Another possible reason underlying the coexistence of lymphopenia and hypercytokinemia is T-cell exhaustion, which is dysfunction of cytotoxic T cells due to an overwhelming immune response. T-cell exhaustion is usually accompanied by prolonged escalated levels of cytokines to compensate for lymphocyte dysfunction [36]. Interestingly, reduced numbers of CD4 + and CD8 + T cells and increased levels of exhaustion-related biomarkers were noted in SARS-CoV-2 patients. These markers included programmed cell death receptor (PD)‐1 and T‐cell immunoglobulin (Ig) and mucin‐domain containing‐3 (Tim‐3) on CD4 + and CD8 + T cells [34] and NKG2A on NK cells and CD8 + T cells [24]. In particular, levels of both PD-1 and Tim-3 on T cells are elevated in SARS-CoV-2 patients from prodromal stages to symptomatic stages [34]. Altogether, we postulated that T-cell apoptosis promoted by immunomodulatory cytokines and T-cell exhaustion due to a prolonged immune response lead to dramatic hypercytokinemia in severe SARS-CoV-2 patients (Fig. 1).

Cytokine storms may lead to unstoppable systemic immune responses and are related to vasodilatory shock, coagulation impairment, multiorgan failure, and even potentially fatal outcomes [37]. The elevated immune response triggered by SARS-CoV-2 may lead to acute pulmonary damage [38] and other extrapulmonary injuries, such as cytokine-induced myocardial dysregulation, stress-related cardiomyopathy [39], and acute liver injury [40] (Fig. 1). It is clinically crucial to determine whether higher levels of cytokines are protective or pathological to SARS-CoV-2-infected patients, especially those with concurrent inflammatory diseases such as CD. In the next section, we look into the potential influence of SARS-CoV-2 regarding the immune response in CD patients.

In SARS-CoV-2-infected patients, lymphocytes such as CD4 + T cells, CD8 + T cells, and natural killer (NK) cells are significantly decreased, which may be partially attributed to increased apoptosis of T cells. In addition, exhaustion-related markers including programmed cell death receptor (PD)-1, T-cell immunoglobulin and mucin domain-containing protein 3 (Tim-3), and NKG2A are increasingly expressed by T cells and NK cells. Meanwhile, cytokine storms, or hypercytokinemia, were also noted in SARS-CoV-2 patients, which included both proinflammatory cytokines and immunomodulatory cytokines, such as interleukin (IL)-2, IL-6, IL-10, and tumor necrosis factor (TNF)-α. The shortage and dysfunction of lymphocytes may be compensated for by elevated levels of cytokines, while increased immunomodulatory cytokines may in turn lead to T-cell apoptosis and T-cell exhaustion. Last, cytokine storms may lead to multiorgan dysfunction, while lymphocytopenia and dysfunction of lymphocytes may predispose SARS-Co-V2 patients to opportunistic infections.

### Impact on Crohn's disease (CD) patients

#### Detection of SARS-CoV-2 in the GI system

Recent meta-analyses showed that the prevalence of GI symptoms in SARS-CoV-2 patients ranged from 10% to nearly 20% with diarrhea as the most common manifestation [41]. Moreover, GI symptoms were more frequent in those with severe SARS-CoV-2 infection (23%) compared to those with mild SARS-CoV-2 infection (8%) [41]. Such higher prevalences encourage investigation of intestinal infection by SARS-CoV-2. It turns out that not only viral RNA and nucleocapsid protein (NP) were found in intestinal biopsies [42], but the permeability of SARS-CoV-2 into the intestinal epithelium was also demonstrated by ex vivo enteroid or organoid models [43]. Furthermore, infectious particles produced by enterocytes and induction of a viral induction program imply that the replication of SARS-CoV-2 can be supported by the intestinal epithelium [44].

#### ACE2: beyond the entry of SARS-CoV-2

##### Different forms and physiological roles of ACE2

Full-length, membrane-bound ACE2 consists of a carboxypeptidase domain, for the formation of angiotensin, and a collectrin domain, for interactions with neutral amino acid transporters. Shed, soluble ACE2 is cleaved from full-length ACE2 by a disintegrin and metallopeptidase domain 17 (ADAM17) or by type II transmembrane serine protease (TMPPSS2), but it still contains an intact carboxypeptidase domain [45]. The carboxypeptidase domain of ACE2 binds with the spike protein of SARS-CoV-2 and the co-expressed TMPRSS2 cleaves the spike protein to facilitate entry of the virus [16]. With the intact carboxypeptidase domain, soluble ACE2 can act as a competitive interceptor of SARS-CoV-2 and therefore hinder its entry [46].

ACE2 expression was found in absorptive enterocytes of the ileum and colon, collected from human single-cell transcriptomes [47], while messenger (m)RNA levels were detected in the duodenum, ileum, cecum, and colon from human donors [48]. Surprisingly, local intestinal inflammation may influence ACE2 expression. ACE2 expression was significantly lower in inflamed ilea collected from CD patients compared to uninflamed samples and was negatively associated with the inflammatory marker, *S100A8* [49]*.* On the other hand, serum levels of both soluble ACE2 and ADAM17 were slightly higher in CD patients compared to patients without an IBD [50]. The decreased transmembrane ACE2 and increased soluble ACE2 may partially explain why the prevalence of SARS-CoV-2 infection is not higher in IBD patients. But confounding factors need to be taken into account, such as compliance with personal hygiene and social distancing; postponement of surgical and endoscopic procedures might be more profound in patients with underlying health concerns [51].

##### The role ACE2 in inflammation in IBD patients

**Fig. 2:**
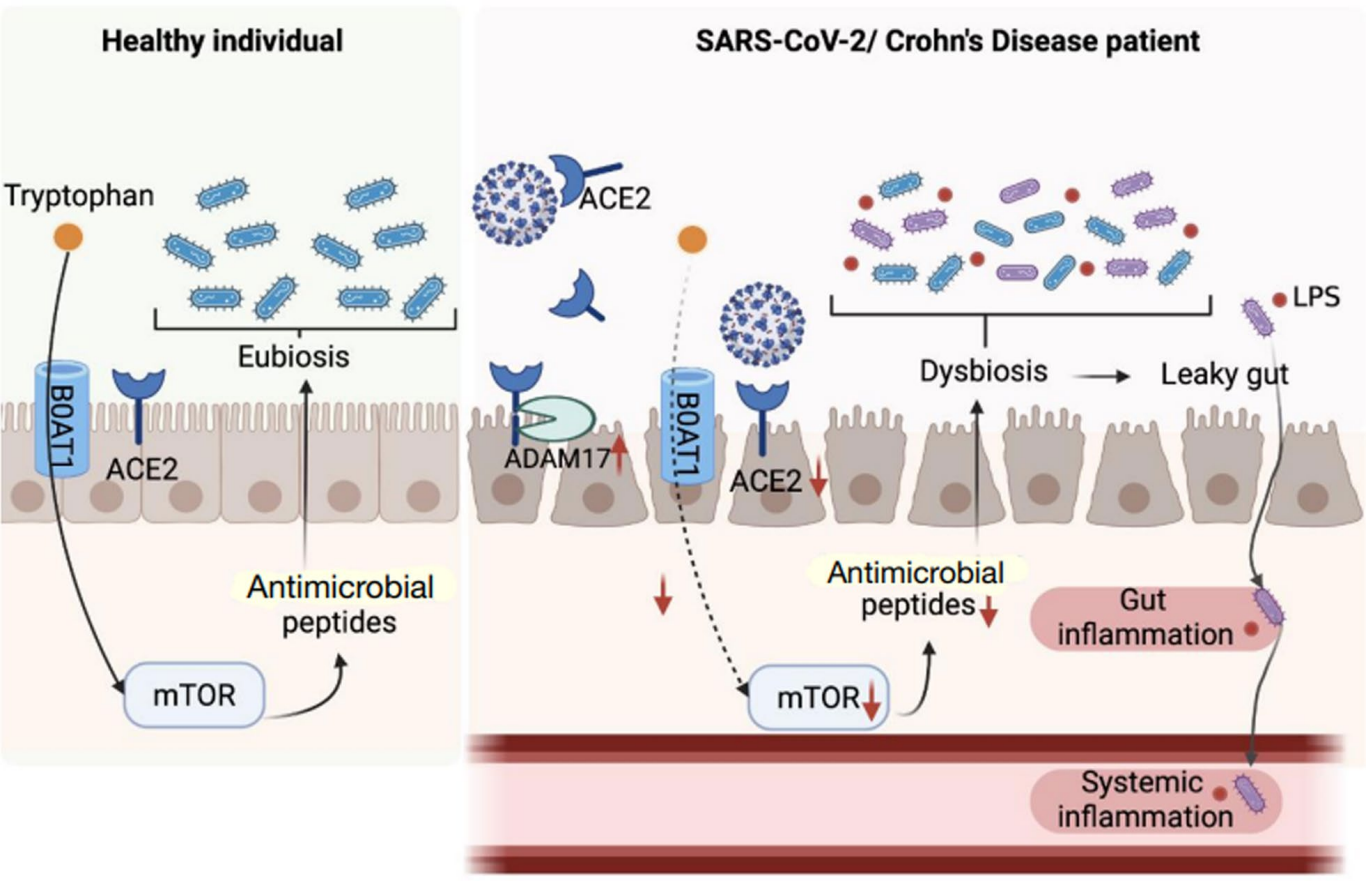
Potential pathogenesis of SARS-CoV-2-related gut inflammation

In the gut, another important function of ACE2 is the transport of the tryptophan amino acid, which is crucial to intestinal immunity and the microbiome. ACE2 stabilizes the amino acid transporter, B0AT1, on the luminal surface of intestinal epithelial cells and therefore facilitates tryptophan absorption. Tryptophan and its metabolite, nicotinamide, regulate expressions of antimicrobial peptides by the mammalian target of rapamycin (mTOR) pathway, which then leads to alterations in the intestinal microbiota [52]. Eventually, disturbances in the microbiotic composition may induce intestinal inflammation. It was reported that *Ace2*-knockout mice with low serum levels of tryptophan developed more-severe colitis and an altered intestinal microbiota [52] (Fig. 2).

In line with lower transmembrane ACE2 expression in CD patients, tryptophan levels were also significantly lower than in healthy controls and were even negatively correlated with CD severity [53]. Furthermore, B0AT1 in colonic biopsies was also significantly lower than in controls [53]. The above findings imply the importance of ACE2 and tryptophan in CD severity. On the other hand, for patients who eventually recover from SARS-CoV-2, the tryptophan level significantly increased during the acute stage of infection [54] but eventually subsided to a level significantly lower than those with ongoing SARS-CoV-2 infection [55]. Tryptophan levels of SARS-CoV-2-positive patients with critical COVID-19 symptoms were significantly lower than those of the SARS-CoV-2-negative group [56]. Given the fact that dysregulated tryptophan metabolism in SARS-CoV-2 may also be attributed to lower tryptophan levels [55], we postulated that there is a dynamic change in serum tryptophan, where tryptophan increases in the early stage of SARS-CoV-2 infection to strengthen antiviral immunity but decreases due to the interruption by SARS-CoV-2 in severe scenarios (Fig. 2). Although determining whether the interaction between SARS-CoV-2 and ACE2 impacts the absorption of tryptophan has not yet been studied, such an interaction might also precipitate local inflammation in CD patients. Future research regarding levels of tryptophan during the COVID-19 progression and their impacts on local inflammation is warranted.

##### ACE2 is associated with altered compositions of the gut microbiota

A lack of ACE2 reduces the production of antimicrobial peptides, which then leads to alterations of the gut microbiota [52], while the composition of the microbiota also influences the intestinal expression of ACE2 [49, 57]. Recent studies showed that the absence of the gut microbiota in several murine models was correlated with increased mRNA levels of *ACE2* [49], whereas the presence of specific gut microbiotic species, such as members of the Bacteroidetes, downregulates ACE2 expression in the murine gut [57]. Strikingly, these Bacteroidetes members are inversely related to the fecal load of SARS-CoV-2 [57], suggesting that the gut microbiota may be associated with susceptibility to SARS-CoV-2 (Fig. 2). In 2023, Bondareva et al. suggested that in addition to affecting ACE2, the microbiota can help the human body fight against SARS-CoV-2. The microbiota induces the body to produce SARS-CoV-2 spike IgG antibodies through molecular mimicry, and as the number of SARS-CoV-2 spike IgG antibodies increases, the abundance of *Streptococcus salivarius* also increases, which is recognized by both SARS-CoV-2 and monoclonal antibodies and accelerates the clearance of SARS-CoV-2, thus increasing the protective immunity of the human body [58]. Therefore, we investigated the composition of the microbiota and discuss possible links between alterations in the microbiota and systemic inflammation in the following section.

#### Gut microbiota, impaired intestinal barrier function, and systemic inflammation

##### Altered composition of intestinal microbiota in CD and SARS-CoV-2 infection

A healthy gut microbiota contributes to inflammatory modulation, intestinal homeostasis, and antiviral immune responses, while gut dysbiosis, an alteration in the gut microbiota, is associated with opportunistic infections, intestinal inflammation, and colorectal cancer [59]. The composition of the gut microbiota is dominated by *Faecalibacterium prausnitzii*, *Eubacterium*, Lachnospiraceae taxa, and Roseburiaare in healthy adults [57], whereas the dysbiotic profile in CD patients features enrichment of pathogenic Enterobacteriaceae [60, 61] decreased anti-inflammatory bacterial species such as *Faecalibacterium prausnitzii* [62], and reduced abundances of short-chain fatty acid-producing bacteria, including members of the Lachnospiraceae and Ruminococcaceae families, which is related to TNF-α antagonist responses [63].

Significant alterations in the composition of the gut microbiota were seen in SARS-CoV-2 patients, which featured lower abundances of anti-inflammatory commensal symbionts, including *Faecalibacterium prausnitzii,* Lachnospiraceae taxa*, Roseburia* spp.*,* and *Alistipes onderdonkii*, and enrichment of opportunistic pathogens, including *Bacteroides nordii*, *Clostridium hathewayi*, and *Actinomyces viscosus* [57]*.* In agreement, another study also showed higher abundances of opportunistic pathogens, such as *Actinomyces, Rothia,* and *Streptococcus* in SARS-CoV-2 patients [64]. Moreover, the composition of the gut microbiota is associated with the severity of SARS-CoV-2 infection. Abundances of *Coprobacillus, Clostridium ramosum,* and *Clostridium hathewayi* were positively correlated, but the abundance of *Faecalibacterium prausnitzii* was negatively correlated with SARS-CoV-2 severity [57]. Strikingly, these alterations in the microbiota persisted even after resolution of respiratory symptoms [57]. An article by Qin Liu et al. noted that the diversity and abundance of the microbiota in patients with post-acute COVID-19 syndrome (PACS) were significantly lower compared to those without PACS or those who had not been diagnosed with COVID-19. In particular, PACS patients had significantly lower levels of *Collinsella aerofaciens*, *Faecalibacterium prausnitzii*, and *Blautia obeum*, and higher levels of *Ruminococcus gnavus* and *Bacteroides vulgatus* compared to those without PACS. In addition, different symptoms in PACS were also reflected in the microbiotic composition of patients; among them, respiratory symptoms (coughing, sputum, nasal congestion/runny nose, and shortness of breath) were positively correlated with a variety of opportunistic pathogens, including *Streptococcus anginosus*, *Streptococcus vestibularis*, *Streptococcus gordonii*, and *Clostridium disporicum*; neuropsychiatric symptoms (headaches, dizziness, loss of taste, loss of smell, anxiety, difficulty concentrating, difficulty sleeping, depression, poor memory, and blurred vision) and fatigue were positively associated with *Clostridium innocuum* and *Actinomyces naeslundii*; abundances of *Bifidobacterium pseudocatenulatum*, *Faecalibacterium prausnitzii*, and *Roseburia inulinivorans* were reduced in patients who had persistent hair loss [65]. Based on the above findings, physiological and pathological impacts of dysbiosis may last beyond the SARS-CoV-2 infection (Fig. 2). Nonetheless, to ascertain causal relationships between the gut microbiome and severity of SARS-CoV-2 infection in future studies, it is critical to consider possible confounding factors that might interfere with the composition of the gut microbiota, such as the use of antibiotics, nutrient intake, comorbidities, and genetic predisposition, and also prospectively monitor the microbial composition at the onset, during the course of, and after recovery from SARS-CoV-2 infection.

##### Dysbiosis may lead to systemic inflammation

The intestinal epithelium acts as a barrier against the entry of pathogenic bacteria, but dysbiosis may disrupt the intestinal barrier by precipitating local inflammation. Once the intestinal barrier is disrupted, gut microbiota may cross the epithelium and induce gut inflammation, and even worse, bacteria and their biological products, such as endotoxins, may pass through the impaired intestinal barrier and translocate to the lymphatic and systemic circulation, causing systemic inflammation [66] (Fig. 2).

Cumulative studies have shown that dysbiosis and bacterial overgrowth are associated with increased intestinal permeability and pathogenesis of CD [67]. An increased level of intestinal absorption and serum levels of lipopolysaccharide (LPS)-binding protein (LBP), CD14, and endotoxin, resulting from increased intestinal permeability, were correlated with CD activity [68, 69]. Moreover, translocation of bacteria from the intestinal lumen to lymph nodes [70] and the bloodstream [71] was discovered in CD patients and may lead to activation of local inflammation and subsequent exacerbation of systemic inflammation [72] (Fig. 2).

As previously mentioned, downregulation of intestinal ACE2 may lead to dysbiosis-induced impairment of the intestinal barrier [52]. The increased permeability may give rise to systemic dissemination of bacterial LPS, peptidoglycan, endotoxins, and metabolites, which may underlie aggravation of GI inflammation and initiation of systemic inflammation and cytokine storms in SARS-CoV-2 patients [73]. Although modulation of the intestinal microbiota appears to be a potential therapeutic option to alleviate systemic inflammation, there are few examples of utilizing an altered microbiota to manage SARS-CoV-2 patients. Lau RI et al. used probiotics to improve post-acute COVID-19 syndrome, including fatigue relief, reduced memory loss, and decreased inattention, dyspepsia, and malaise. The probiotic group also showed higher bacterial diversity in stool samples, including species of the *Bifidobacterium* genus (*B. bifidum*, *B. adolescentis*, *B. longum*, and *B. pseudocatenulatum*), *Roseburia intestinalis*, *Roseburia* spp., *Roseburia hominis*, *Faecalibacterium prausnitzii*, and *Akkermansia muciniphila*,[changes OK??] and the study also indicated that attentional remission was associated with an increased abundance of *Bifidobacterium longum* [74]. Wischmeyer et al. found that the use of the probiotic, *Lacticaseibacillus rhamnosus* GG (LGG), as a prophylactic agent after exposure to SARS-CoV-2 significantly reduced infection rates, and the abundance of *Lacticaseibacillus rhamnosus* in feces of subjects taking probiotics was also significantly increased. Moreover, the microbial structure (i.e., β-diversity) of feces also significantly differed [75]. Nevertheless, more applications for altering the microbiota remain to be explored. Therefore, to tackle systemic inflammation, immunosuppressive medication has been administered for both CD and SARS-CoV-2 patients. In the next section, we discuss the pros and cons of immunosuppressants for CD in the context of SARS-CoV-2 infection.

Angiotensin-converting enzyme 2 (ACE2) stabilizes B0AT2 transporters and therefore facilitates the intestinal absorption of tryptophan. Tryptophan-stimulated mammalian target of rapamycin (mTOR) promotes the production of antimicrobial peptides, which benefits modulation of the gut microbiota in healthy individuals. In SARS-CoV-2-infected or Chron’s disease (CD) patients, decreased expression of transmembrane ACE2 due to cleavage by a disintegrin and metallopeptidase domain 17 (ADAM17) or interaction with SARS-CoV-2 may interrupt the absorption of tryptophan, which potentially leads to dysregulation of the gut microbiota. Dysbiosis can trigger leaky gut syndrome, increasing the permeability to pathogens and lipopolysaccharide (LPS), which then aggravates local and systemic inflammation. Image created using Biorender.

## Immunosuppressants

The severity of CD determines the clinical approaches and is defined by the Crohn's Disease Activity Index [76]. For those with mild CD, step-up therapy is suggested whereby oral glucocorticoids are usually given prior to other more-potent drugs. Additionally, maintenance therapy with glucocorticoids should be tapered down to avoid steroid-induced side effects. In contrast, patients with moderate to severe CD are initially treated with immunomodulators or biologics in a top-down manner, where TNF inhibitors are given with or without immunomodulators (azathioprine, 6-mercaptopurine, or methotrexate) prior to other alternatives including an anti-IL-12/23 antibody and an anti-integrin antibody. Since the pros and cons of immunosuppressive agents on SARS-CoV-2 infection have been one of the most debatable issues, here, we summarize SARS-CoV-2 outcomes of IBD patients with each immunosuppressive agent and organize the basic information of references in Table 1.

**Table 1:** Summary of basic information of references, including types of articles, geographical distribution, time information, number of cases, and vaccine type

	Type of article	Geographical distribution	Time information	No. of cases	Vaccine type	Ref.
Steroids	Case report	Brazil	2020	1	None	[78]
	Cohort study	Italy	No data available	79	None	[79]
	Cohort study	United States	2020/1/20–2020/12/10	30,911	None	[80]
	Cohort study	Europe	2020/2/21–2020/6/30	23,879	None	[81]
	No data available	Worldwide	No data available	525	None	[82]
	Clinical trial	UK	No data available	2014	None	[83]
TNF antagonist	Letter to the editor	Italy	No data available	2	None	[86]
	Cohort study	Worldwide	2020/1/1–2020/5/15	37,875	None	[88]
	Cohort study	Italy	2020/3/11–2020/3/29	79	None	[79]
	Cohort study	Worldwide	2020/3/13–2020/6/9	1439	None	[7]
	No data available	France	2020 2/15–2020 8/31	26,800	None	[90]
	Cohort study	No data available	2020/2/15–2020/8/31	6144	None	[89]
	Cohort study	UK	2020 9/22–2020/12/23	6935	None	[91]
	Postscript letter	UK	2020/5–2020/12	No data available	None	[92]
	No data available	No data available	No data available	1293	BNT162b2, ChAdOx1nCoV-1	[93]
	No data available	UK	2020/9/22–2020/12/23	7226	BNT162b2, ChAdOx1 nCoV-1	[94]
Thiopurine	Cohort study	Worldwide	2020/1/1–2020/5/15	37,875	None	[88]
	Cohort study	Italy	2020/3/11–2020/3/29	79	None	[79]
	No data available	France	2020 2/15–2020 8/31	26,800	None	[90]
	Cohort study	Worldwide	2020/3/13–2020/6/9	1439	None	[7]
	No data available	No data available	No data available	1293	None	[97]
	No data available	UK	2020/9/22–2020/12/23	7226	None	[94]
Anti-α4/β7 antibody	Cohort study	United States	2020/1/20–2020/12/10	30,911	None	[80]
	No data available	France	2020 2/15–2020 8/31	26,800	None	[90]
	Cohort study	No data available	2020/2/15–2020/8/31	6144	None	[89]
Anti-IL12/23 antibody	Meta-analysis	Worldwide	2019/12–2021/8	No data available	None	[101]
	Cohort study	No data available	No data available	1909	1123 BNT162b2, 692 mRNA-1273 94 Ad26.COV2.S	[102]

*Ref.* reference, *TNF* tumor necrosis factor

### Corticosteroids

Corticosteroids, including prednisone and budesonide, are commonly used to initiate symptom relief for moderate to severe CD patients for a short period of time to avoid long-term side effects [77]. For patients infected with SARS-CoV-2, prednisone should be tapered to less than 20 mg/day or substituted with budesonide, but once COVID-19 is confirmed, all systemic corticosteroids should be discontinued [8].

In a young female with severe CD and SARS-CoV-2 infection without oxygen supplementation, maintenance with prednisone, a TNF-α inhibitor, and adalimumab showed favorable outcomes of the SARS-CoV-19 infection with no exacerbation of CD [78]. Another prospective observational cohort study including 79 IBD patients (32 CD and 47 ulcerative colitis (UC) patients) showed that concomitant corticosteroid treatments were not associated with the risk of SARS-CoV-2-related pneumonia or death [79].

However, multiple cohort studies showed unfavorable results regarding corticosteroid treatments of IBD patients with SARS-CoV-2 infection. In a retrospective cohort study examining 30,911 IBD patients (51.2% with CD and 58.8% with UC), corticosteroid use was associated with increased hazard ratios of SARS-CoV-2 infection and SARS-CoV-2-related hospitalization and mortality compared to those without corticosteroid treatment [80]. Similarly, another prospective, observational, cohort study recruiting 23,879 IBD patients, among whom 53 CD and 43 UC were infected with SARS-CoV-2, showed that corticosteroid treatments were associated with an increased risk of hospitalization (odds ratio (OR) 7.69, 95% confidence interval (CI) 1.48–40.05) [81]. Finally, an international registry collecting 525 IBD patients (59.4% with CD and 38.7% with UC) also reported that corticosteroid use was associated with an increased OR of admission to an intensive care unit (ICU), ventilator use, or hospitalization [82]. Although corticosteroid treatment was associated with severe SARS-CoV-2 outcomes, corticosteroid use was not necessarily the cause. One plausible explanation is that severe underlying conditions predispose IBD patients to hospitalization and the urgency of corticosteroid use, which is consistent with the RECOVERY trial whereby only severe SARS-CoV-2 patients requiring ventilation benefited from corticosteroid use [83].

### TNF antagonists

TNF antagonists block interactions between TNF-α and TNF-α receptors and therefore hinder the TNF-α-mediated proinflammatory signaling pathway and expressions of inflammatory genes [84]. TNF antagonists, including infliximab, adalimumab, and certolizumab pegol, are widely used as monotherapy or combination therapy with thiopurine to treat CD [85]. However, TNF antagonists should be delayed for 2 weeks upon infection with SARS-CoV-2 and even discontinued during acute illness due to COVID-19 [8].

Administration of TNF antagonists seemed to have positive outcomes in both adult and pediatric cases. A 30-year-old CD patient treated with the TNF-α inhibitor, adalimumab, was infected by SARS-CoV-2 but recovered shortly after mild pneumonia without recurrence of CD [86]. Similarly, another TNF-α inhibitor, infliximab, also effectively treated both moderate to severe CD and SARS-CoV-2-related multisystem inflammatory syndrome in pediatric CD patients with recent SARS-CoV-2 infections [87].

Multiple cohort studies have suggested that monotherapy of TNF antagonists was not associated with adverse clinical outcomes. In a retrospective cohort study that recruited 37,857 IBD patients, among whom 36 developed incident COVID-19, TNF antagonists were not associated with an increased risk of SARS-CoV-2 infection [88]. Likewise, in another prospective observational cohort study including 79 IBD patients (32 CD and 47 UC patients), concomitant TNF antagonists were not associated with SARS-CoV-2-related pneumonia or death [79]. Consistently, reports from an international registry showed that administration of TNF antagonists to IBD patients was not associated with severe SARS-CoV-2 outcomes, such as admission to an ICU, ventilator use, hospitalization, or death [7, 82, 89].

Nonetheless, conflicting findings regarding combination therapy of TNF antagonists with thiopurine were found in different cohort studies. A study from an international registry of over 1400 IBD patients suggested that combined therapy of a TNF antagonist and thiopurine was associated with an increased risk of severe SARS-CoV-2 outcomes, defined as a composite of mechanical ventilation, ICU admission, or death [7]. Further study from an international registry including more than 6000 IBD patients reported that the combination of a TNF antagonist and thiopurine was associated with a significantly increased risk of hospitalization or death but not with severe SARS-CoV-2 outcomes [89]. Conversely, another nationwide, unselected, population-based study of 600 SARS-CoV-2 patients among over 268,000 IBD patients showed that among SARS-CoV-2-infected IBD patients, neither monotherapy nor combined therapy with a TNF antagonist was associated with an increased risk of hospitalization compared to those without treatment [90]. These conflicting findings may have resulted from different reporting systems, whereby over-reporting of patients receiving multiple medications is more likely to be introduced in physician-reported studies.

Mechanistically, infliximab seems to directly influence the serologic response, which may explain the association between combination therapy and severe SARS-CoV-2 outcomes reported in multiple large-scale studies [7, 89]. In terms of serological responses of IBD patients to SARS-CoV-2 infection, one study showed that seroconversion, seroprevalence, and antibody reactivity were lower in IBD patients treated with infliximab compared to vedolizumab, with the lowest antibody responses in patients treated by infliximab combined with thiopurines or methotrexate [91]. Furthermore, another study showed that the combination of infliximab and thiopurine was associated with significantly reduced neutralizing antibody responses and SARS-CoV-2-related immunoglobulin responses, including IgA spikes, IgG spikes, IgG receptor-binding domain of the spike, and IgG nucleocapsid responses, in IBD patients compared to healthy controls [92]. Surprisingly, infliximab treatment not only influenced serological responses but may also have attenuated immunogenicity in IBD patients following the first and second doses of SARS-CoV-2 vaccines. Studies showed that anti-SARS-CoV-2 spike antibody concentrations following first and second vaccinations in IBD patients treated with infliximab were lower than with vedolizumab. Lower rates of seroconversion, shorter half-lives of antibodies, and an increased risk of a breakthrough infection were also found in patients treated with infliximab compared to vedolizumab [93, 94]. Altogether, it is increasingly imperative to consider whether anti-TNF monotherapy and combination therapy outweigh the potential risks of attenuated serological responses, which may increase susceptibility to recurrent SARS-CoV-2 infections at an individual level and lead to chronic viral colonization and consequently prolonged transmission in populations. However, the underpinning mechanism of decreased antibody responses remains elusive.

### Thiopurines

Thiopurines inhibit nucleic acid synthesis in dividing cells and thus hinder the clonal proliferation of the adaptive immune response [95]. For CD patients, thiopurines, including azathioprine, mercaptopurine, and thioguanine, are given in combination with TNF antagonists or as alternative monotherapy for patients who have received glucocorticoids or have had no response to biologics [96]. Nonetheless, thiopurines should be temporarily suspended upon infection with SARS-CoV-2 and even discontinued during acute COVID-19 illness [8].

Two cohort studies showed that concomitant thiopurine treatments were not associated with an increased risk of SARS-CoV-2 infection [88], SARS-CoV-2-related pneumonia, death [79], or hospitalization [90] among IBD patients. Nonetheless, a contradictory result was reported by an international registry where thiopurine monotherapy and combination therapy with a TNF antagonist in IBD patients were associated with an increased risk of severe SARS-CoV-2 outcomes, encompassing ICU admissions, mechanical ventilation, or death [7]. Regarding this controversy, besides the over-reporting bias in the physician-reported study mentioned earlier, the under-reporting of patients with mild symptoms or without medication is possibly contributory as well. Last, the attenuation of anti-SARS-CoV-2 antibody responses was less remarkable compared to IBD patients treated with infliximab [93, 94].

### Anti-alpha4/beta7 antibody

The anti-alpha4/beta7 antibody blocks interaction between α_4_β_7_ integrin and MAdCAM-1 and therefore prevents the trafficking of immune cells and mucosal inflammation of the GI tract [97]. Vedolizumab, one of the alpha4/beta7 antibodies, is used as monotherapy for first-line biologic therapy but is used in combination with an immunomodulator for second- or third-line therapy for active moderate to severe CD patients [76, 98]. For SARS-CoV-2 infected patients, vedolizumab should be delayed for 2 weeks while monitoring the onset of COVID-19. Once COVID-19 is confirmed, discontinuation of vedolizumab is necessary [8].

Among different IBD treatments, vedolizumab is associated with an increased risk of SARS-CoV-2 infection compared to mesalazine but did not significantly differ from anti-TNF regarding combined outcomes of SARS-CoV-2-related hospitalization and mortality in a retrospective cohort study of 30,911 IBD patients [80]. In terms of the risk of SARS-CoV-2-related hospitalization and mortality, one unselected, population-based study showed no difference in IBD with or without vedolizumab [90], while another study from a physician-reported international registry showed that vedolizumab treatment was associated with decreased risks [89].

### Anti-IL12/23 antibody

Ustekinumab is a monoclonal antibody that blocks the p40 subunit of IL-12 and IL-23 receptors on T cells, NK cells, and antigen-presenting cells [99]. Ustekinumab monotherapy is usually administered to CD patients without prior biologic exposure. Additionally, Ustekinumab in combination with an immunomodulator such as thiopurine is used for CD patients who failed at least one biologic, monoclonal antibody, or corticosteroid treatment [76, 100]. As for SARS-CoV-2-infected patients, Ustekinumab should be suspended for 2 weeks or discontinued during acute COVID-19 illness [8].

In terms of severe SARS-CoV-2 outcomes, including ICU admission, ventilator use, and SARS-CoV-2-related death, an international registry first showed that Ustekinumab did not significantly differ from TNF antagonist monotherapy [7] and further revealed that both Ustekinumab and a TNF antagonist were associated with lower risks [89]. Similarly, a systematic review and meta-analysis showed that Ustekinumab and TNF antagonists were associated with decreased odds of SARS-CoV-2–related hospitalization or death [101]. Last, Ustekinumab was associated with decreased odds of lacking an antibody response among IBD patients who had received a SARS-CoV-2 vaccination [102].We summarize treatment for patients with Crohn’s disease and COVID-19the basic information of references in Table 2. We Summary from cohort studies regarding COVID-19 outcomes of inflammatory bowel disease patients treated with immunosuppressants in Table 3.

**Table 2:** Summary of treatment for patients with Crohn’s disease and COVID-19

	Drug	Mechanism	Expert comments	Ref.
Steroids	Prednisone	Systemically suppresses immune responses	Prednisone should be tapered to less than 20 mg/day or substituted with budesonide	[8]
	Budesonide		Suspend the treatment until the resolution of COVID-19	
TNF antagonists	Infliximab, Adalimumab Certolizumab pegol	Hinders the TNF-α-mediated signaling pathway	Delay treatment for 2 weeks once infected with SARS-CoV-2	[8, 84, 103, 104]
			Suspend treatment until the resolution of COVID-19	
Thiopurines	Azathioprine, Mercaptopurine	Inhibits nucleic acid synthesis during proliferation of adaptive immune cells	Delay treatment for 2 weeks once infected with SARS-CoV-2	[8, 95]
	Thioguanine		Suspend treatment until the resolution of COVID-19	
Anti-α4/β7 antibody	Vedolizumab	Hinders the trafficking of immune cells by blocking the interaction between α4β7 integrin and MAdCAM-1	Delay treatment for 2 weeks once infected with SARS-CoV-2	[8, 97]
			Suspend treatment until the resolution of COVID-19	
Anti-IL12/23 antibody	Ustekinumab	Blocks the p40 subunit of IL-12 and IL-23 receptors on T cells, natural killer cells, and antigen presenting cells	Delay treatment for 2 weeks once infected with SARS-CoV-2	[8, 99]
			Suspend treatment until the resolution of COVID-19	

*Ref.* reference, *TNF* tumor necrosis factor

**Table 3:** Summary from cohort studies regarding COVID-19 outcomes of inflammatory bowel disease patients treated with immunosuppressants

	Favorable	Unfavorable	Ref.
Steroids	No data available	Associated with an increased risk of SARS-CoV-2 infection and SARS-CoV-2-related hospitalization [80, 81], mortality [80], and severe COVID-19 outcomes [82]	[80–82]
TNF antagonist	Monotherapy was not associated with an increased risk of SARS-CoV-2 infection [88], SARS-CoV-2-related pneumonia [79], hospitalization [90], severe COVID-19 outcomes [7, 82, 89], or death [79]	No available data for monotherapy	[7, 79, 82, 88–90, 92]
		Combination therapy of a TNF antagonist and thiopurine was associated with an increased risk of severe COVID-19 outcomes [7], hospitalization or death [89], lower seroconversion, seroprevalence, antibody reactivity [91], and immunoglobulin responses against SARS-CoV-2 [92]	
	Combination therapy of TNF antagonists was not associated with an increased risk of hospitalization [90]		
Thiopurine	Not associated with an increased risk of SARS-CoV-2 infection [88], SARS-CoV-2-related pneumonia, death [79], or hospitalization[90]	Combination therapy of thiopurine and a TNF antagonist was the same as above	[79, 88, 90]
Anti-α4/β7 antibody	Not associated or inversely associated with increased risk of SARS-CoV-2-related hospitalization or mortality [89, 90]	Associated with an increased risk of SARS-CoV-2 infection [80]	[80, 89, 90]
Anti-IL-12/23 antibody	Associated with lower risks of severe COVID-19 outcomes [89], hospitalization, or death [101], and decreased odds of lacking an antibody response against SARS-CoV-2 [102]	No data available	[89, 101, 102]

*Ref.* reference, *TNF* tumor necrosis factor

## Conclusions

This article first discussed how lymphocyte counts increase in the acute stage of SARS-CoV-2 infection but decrease during disease progression in patients who eventually died of COVID-19. Although the detailed mechanism of lymphocytopenia is not well understood, we proposed two possible reasons: a higher frequency of T-cell apoptosis and higher levels of immunomodulatory cytokines in SARS-CoV-2-infected patients. T-cell apoptosis or exhaustion may contribute to rebound cytokine storms, which lead to systemic inflammation in SARS-CoV-2-infected patients. As for CD, lower transmembrane ACE2 expression might partially explain why SARS-CoV-2 infection is not increased compared to the general population, and also why it is associated with impaired tryptophan absorption and a subsequently altered composition of the microbiota, which may lead to impairment of the intestinal barrier. Such disrupted levels of tryptophan and dysbiosis of the gut microbiota were found in SARS-CoV-2-infected patients as well, but whether these factors exacerbate systemic inflammation and cytokine storms in CD patients has not yet been studied. Last, we summarize the effects of immunosuppressants, including corticosteroids, TNF antagonists, thiopurines, anti-integrin antibodies, and anti-IL-12/23 antibodies, on CD patients with concurrent SARS-CoV-2 infection in terms of clinical outcomes, serological responses to SARS-CoV-2, and immunogenicity of SARS-CoV-2 vaccines.

This article not only reexamines possible mechanisms of SARS-CoV-2-induced immune responses, but also provides insights for physicians to carefully select immunosuppressive agents in CD patients who also have a SARS-CoV-2 infection. However, additional factors that affect the microbiota and their mechanisms remain to be clarified, and relationships among changes in the microbiota, human immunity, and conditions of CD patients also need to be studied. In addition, SARS-CoV-2 is rapidly mutating. New omicron variants have been spreading globally since the end of 2021, and new subvariants continue to appear. In the summer of 2022, subvariants BA.4 and BA.5 became the most common infectious strains globally, and in January 2023, a new omicron subvariant, XBB.1.16, emerged and was labeled as “one to watch” by the WHO. Although relevant organizations have been actively searching for new therapies and vaccines, the effects of SARS-CoV-2 on various human systems are still unclear. In the future, whether different strains of the virus will affect CD patients differently is a question that still needs to be investigated, and whether the speed of research can keep up with the fast-changing virus is also a major challenge for scientists.

## Data Availability

No datasets were generated or analysed during the current study.
